# From grammar to sustainability: A corpus-informed pedagogical model (GSIC) based on African Leaders’ UNGA-2025 English speeches

**DOI:** 10.1371/journal.pone.0355457

**Published:** 2026-08-11

**Authors:** Olusiji Adebola Lasekan, Margot Teresa Godoy Pena, Blessy Sarah Mathew

**Affiliations:** 1 Departamento de Educación e Innovación, Universidad Católica de Temuco, Temuco, Chile; 2 Languages Coordination, DITFO, Universidad de La Frontera, Temuco, Chile; 3 Department of Economics, Lovely Professional University, Phagwara, Punjab, India; Shandong University, CHINA

## Abstract

This study investigates how African leaders linguistically construct sustainability discourse in their United Nations General Assembly (UNGA-2025) speeches and demonstrates how these linguistic patterns can be transformed into a pedagogical model for Education for Sustainable Development (ESD). A 50,644-token corpus of 21 African leaders’ UNGA-2025 English speeches was analyzed across five linguistic levels—lexical, morphological, syntactic, pragmatic, and discourse—using a mixed quantitative–qualitative approach. Results show that sustainability meaning is built predominantly through lexical items (48.6%) and discourse organization (26.5%), supported by pragmatic strategies (16.5%) emphasizing urgency and collective agency. Key grammatical resources—including modal verbs (must, should), conditional statements, passive constructions, collective pronouns, and problem–solution sequencing—map systematically onto sustainability competencies: normative, systems-thinking, interpersonal, anticipatory, and strategic. Strategic and interpersonal competencies dominated, reflecting Africa’s emphasis on governance, multilateral partnership, innovation, and climate action. Based on these mappings, the study proposes the Grammar-for-Sustainability Instructional Cycle (GSIC), a five-phase task model (noticing, analysis, transformation, production, reflection) that operationalizes authentic UNGA discourse for language instruction. GSIC supports simultaneous development of grammatical control and sustainability competencies, positioning grammar as purposeful social action. The findings contribute a novel linguistic–competency mapping framework and demonstrate how corpus-informed pedagogy can enrich ESD, particularly within African English language education.

## 1. Introduction

The relationship between language, sustainability, and education is a multifaceted and evolving field that integrates linguistic skills with the goals of sustainable development to foster global citizenship, cultural understanding, and environmental awareness. Increasingly, language education—particularly in widely used languages such as English and Mandarin—has been positioned as a platform for engaging learners with sustainability challenges by embedding SDG-aligned content into curriculum and instruction. Research in English Language Teaching (ELT) demonstrates that sustainability principles can be infused into classroom practice to cultivate ecological consciousness and global citizenship [1,2], as seen in studies examining SDG-integration in Thai English textbooks [3]. Similar work in Mandarin education highlights interdisciplinary and project-based learning as strategies for promoting global citizenship and intercultural understanding [4]. Technology-enhanced strategies—such as virtual learning environments—further support sustainability learning by enabling dynamic content access and meaningful participation [5], while open educational resources and teacher training help address challenges related to curricular alignment and limited materials [2]. Despite growing interest, empirical studies assessing the classroom impact of such approaches remain limited [6], research remains geographically concentrated, and the role of language in Education for Sustainable Development (ESD) across many regions, particularly Africa, continues to be underexplored—highlighting the need to incorporate diverse linguistic and cultural knowledge systems, including indigenous languages, into sustainable education frameworks [7].

The United Nations General Assembly (UNGA) speeches function as a key arena for international sustainability discourse, offering insight into how world leaders frame global priorities and articulate national commitments to sustainable development. As highly visible, diplomatically consequential texts, these speeches provide rich linguistic and thematic data through which the authenticity of sustainability engagement can be examined. Analyses of UNGA rhetoric reveal how leaders mobilize specific linguistic and discursive strategies—including topicalization, metaphor, and narrative positioning—to construct ideological perspectives on global issues; for example, Imran Khan’s speeches foreground peace and counter-Islamophobia through strategic framing [8], while Shahbaz Sharif emphasizes harmony to advance national positioning [9]. Sustainability themes frequently intersect with climate change, development, and geopolitical relations, echoing the SDGs’ emphasis on universality and shared responsibility [10]. At the same time, sustainability discourse is embedded in broader policy reform narratives, its conceptual flexibility enabling both substantive commitments and rhetorical appropriation; as such, scholars argue that sustainability can function as an “empty signifier,” strategically reshaped within political debates [11]. Narrative and polyphonic analyses confirm that sustainability messaging varies across contexts—sometimes reinforcing global consensus, other times diverging sharply—demonstrating the dynamic, contested, and ideologically charged nature of UNGA sustainability discourse [12].

Grammar plays a pivotal role in shaping sustainability competencies by enabling effective communication, fostering critical and systems thinking, and supporting intercultural understanding—skills essential for engaging with global sustainability challenges. When integrated into language education, grammatical instruction becomes a vehicle for developing key competencies such as anticipatory, systems-thinking, normative, strategic, and interpersonal skills. Research demonstrates that carefully designed grammatical activities—such as work with passive voice, pronouns, and conjunctions—can deepen learners’ ability to analyze and communicate sustainability issues [13]. Such grammar-supported development aligns with the cultivation of transversal competencies, which prepare students to navigate complex socio-environmental challenges [14]. Broader language-based approaches reinforce this connection: linguistic determinism highlights how language shapes perception and action, supporting the translation of SDGs into inclusive and equitable practice [15], while eco-linguistics reveals how ecocentric language choices can foster environmental responsibility and interconnectedness [16]. Efforts to build lexical ecological literacy—such as teaching names of species and natural phenomena—further empower learners to understand and communicate ecological concerns [17]. Together, this scholarship underscores grammar’s central role in developing sustainability competencies within language education.

Despite growing recognition of the interconnectedness between language education and sustainability, a significant gap persists in the corpus-driven integration of authentic sustainability discourse into grammar pedagogy. Current research highlights promising directions—such as SDG-aligned textbook analysis, interdisciplinary and project-based language instruction, and the use of eco-linguistic perspectives—yet these efforts remain largely conceptual, context-specific, or limited to thematic rather than linguistic integration. While UNGA speeches represent a rich, authentic repository of sustainability discourse characterized by distinctive grammatical, pragmatic, and discursive patterns, studies rarely operationalize this corpus to inform classroom grammar instruction or link linguistic forms to UNESCO sustainability competencies. Most pedagogical work focuses on general SDG awareness, intercultural engagement, or thematic exposure, without explicitly connecting functional grammatical features—such as modal obligation, conditional reasoning, or collective pronoun use—to the development of anticipatory, normative, strategic, systems-thinking, and interpersonal competencies. Moreover, empirical applications remain scarce, particularly in African contexts where ESD and corpus-based language pedagogies are underrepresented. Consequently, there is a pressing need for research that systematically analyzes sustainability discourse at multiple linguistic levels and translates these findings into pedagogical models that empower learners to construct sustainability-aligned meanings through grammar, bridging the gap between corpus evidence and instructional practice.

The purpose of this study is to examine how sustainability discourse can be linguistically operationalized to support Education for Sustainable Development (ESD) by drawing on authentic United Nations General Assembly (UNGA) speech data. First, the study seeks to identify the grammatical features that characterize sustainability discourse across multiple linguistic levels—lexical, morphological, syntactic, pragmatic, and discourse (RO1). Second, it aims to map these linguistic patterns to UNESCO sustainability competencies—including anticipatory, systems-thinking, normative, strategic, and interpersonal competences—to determine how specific grammatical forms (e.g., modal verbs, conditionals, collective pronouns, problem–solution structures) function as vehicles for sustainability meanings (RO2). Building on these empirical findings, the study’s final objective is to develop and justify a Grammar-for-Sustainability Instructional Cycle (GSIC), a task-based pedagogical model that operationalizes corpus-derived linguistic features into instructional practice to foster both grammatical development and sustainability competencies in language learners (RO3). Through this three-stage inquiry, the study addresses the gap between authentic sustainability discourse and classroom grammar instruction, offering an evidence-based framework that positions grammar as purposeful social action aligned with the Sustainable Development Goals.

## 2. Literature review

Grammar plays a crucial role in meaning-making by shaping how stance, obligation, and agency are encoded and interpreted across linguistic contexts. At the level of stance, lexico-grammatical resources such as modal verbs and stance adverbs enable speakers to convey attitudes and evaluations, with discourse genres like reviews and opinion blogs frequently employing such grammatical stance devices [18]; even subtle grammatical shifts—as in the progressive aspect of *believe*—can add immediacy and intensity, demonstrating the pragmatic depth of stance construction [19]. Grammar likewise structures obligation through modality, where forms such as *must* or *should* express varying degrees of necessity and reflect social norms embedded in discourse [20]. These grammatical choices also index ideological positioning, linking obligation to broader socio-cultural expectations. Agency is similarly mediated through grammar: speakers strategically deploy grammatical forms, such as non-anaphoric references, to assert control, claim responsibility, or foreground particular actors [21], a function increasingly salient as communicative authority becomes more distributed in contemporary discourse [22]. While lexical-syntax provides the structural basis for explicit message construction [23], meaning-making extends beyond sentence-level grammar into pragmatics and discourse, where implicit meaning, contextual inference, interactional positioning, and ideological framing emerge [24]. Thus, grammar is not merely a system of formal rules, but a dynamic resource that interacts with pragmatic and discursive processes to construct social meaning.

Corpus linguistics has transformed language education by advancing corpus-informed pedagogy, in which authentic language data serve as the basis for instruction and learning. Rooted in foundational work by Sinclair and Hunston, this approach promotes data-driven learning (DDL) whereby learners investigate concordance lines to observe recurrent linguistic patterns, fostering autonomy, language awareness, and deeper understanding of form–meaning relationships [25,26]. Within this paradigm, teachers act as mediators who guide exploration and scaffold learners’ analytical skills [26], while corpus-based descriptions provide empirically grounded models of usage that can be directly applied to instruction [27]. A central technique is concordance-based noticing, which enables learners to examine language in context, identify collocations, and discern subtle distinctions in meaning and function—skills that support the development of linguistic sensitivity and pragmatic competence. The DDL environment encourages learner agency and motivation by positioning students as researchers who construct their own insights, although challenges such as limited teacher preparation and the technical complexity of corpus tools remain [28]. Targeted scaffolding, task design, and peer collaboration have been proposed as strategies to overcome these constraints. Overall, corpus-informed pedagogy enriches language education by combining authenticity, contextual exposure, and inquiry-driven learning processes that align with contemporary communicative and constructivist instructional goals.

The integration of language education and sustainability, particularly through the lens of Education for Sustainable Development (ESD), has emerged as a promising yet underexplored area, with UNESCO-ESD frameworks emphasizing the development of key sustainability competencies—anticipatory, systems-thinking, normative, strategic, and interpersonal—that can be meaningfully supported through language learning. Studies demonstrate that language classrooms can cultivate these competencies by embedding SDG-related themes into communicative activities and textbook design, as seen in the Headway EFL series where grammatical exercises are intentionally linked to sustainability topics [13], and in communicative English classrooms shown to foster SDG awareness and engagement [29]. Transformative language teaching for sustainability (TLS) further highlights learner-centered, reflective pedagogies aimed at empowering students to address sustainability challenges [30]. Because communication mediates knowledge-sharing, collaboration, and cultural understanding, language-based approaches play a central role in translating SDGs into action, promoting inclusivity, equity, and global citizenship [15,31], supported by teacher training programs that equip educators to integrate sustainability into instruction (Mason et al., 2024). However, despite these advances, research explicitly connecting ESD and grammar remains limited; existing work suggests that grammatical features—such as passive constructions, pronouns, and conjunctions—could serve as linguistic vehicles for sustainability competencies [13]. Broader textbook analyses, such as SDG integration in Thai English materials, point to uneven sustainability representation [3], underscoring the need for systematic models linking grammar instruction to sustainability-oriented competencies.

The United Nations General Assembly (UNGA) has become a primary arena for authentic sustainability discourse, especially following the global shift from the Millennium Development Goals (MDGs) to the Sustainable Development Goals (SDGs), which reframed international development priorities around long-term sustainability rather than short-term development outcomes. This paradigm shift has driven a transformation in global communication—from development communication to sustainability communication—anchoring sustainability as the central theme of UNGA discussions and policy negotiations [32]. UNGA speeches and SDG-related texts consistently highlight the interconnectedness of environmental protection, social equity, and economic development, using cohesive linguistic framing to present the SDGs as a universal, urgent call to action requiring shared responsibility across nations [10]. As a diplomatic and deliberative space, the UNGA mediates the political and institutional dynamics of SDG implementation, facilitating multilateral dialogue, collaborative policymaking, and flexible responses to global challenges [33]. Discussions increasingly integrate climate and environmental concerns into development debates, signaling a broader shift toward holistic sustainability orientations and the mobilization of cross-sectoral participation [34]. Collectively, this scholarship positions the UNGA as a crucial platform where sustainability narratives are negotiated and legitimized, making its discourse a valuable corpus for examining how global actors linguistically construct sustainable futures.

This study is guided by three interrelated research questions. First, it asks what grammatical features characterize sustainability discourse across multiple linguistic levels—lexical, morphological, syntactic, pragmatic, and discourse—in UNGA speeches. Second, it investigates how these linguistic patterns map onto UNESCO sustainability competencies—including anticipatory, systems-thinking, normative, strategic, and interpersonal competencies. Finally, it examines how these corpus-derived insights can be transformed into a pedagogical framework—the Grammar-for-Sustainability Instructional Cycle (GSIC)—that integrates grammatical instruction with SDG-aligned learning to promote both linguistic development and sustainability competencies in language learners. Collectively, these questions seek to connect linguistic description with pedagogical practice, establishing an empirical foundation for corpus-informed sustainability grammar instruction.

## 3. Conceptual framework

African leaders’ UNGA-2025 speeches articulate the continent’s sustainability priorities—highlighting peace, justice, innovation, climate action, and social equity—and demonstrate how sustainability discourse is linguistically constructed through lexical, morphology, syntax, pragmatic and discourse framing to signal urgency, shared responsibility, and proactive policy vision. Corpus analysis shows that these grammatical features map directly onto sustainability competencies: normative, systems-thinking, interpersonal, anticipatory, and strategic competence. Because SDG competencies are grammatically encoded, African sustainability discourse offers an authentic blueprint for pedagogical innovation, suggesting that grammar instruction can be redesigned to foster sustainability competencies. Accordingly, the Grammar-for-Sustainability Instructional Cycle (GSIC) is proposed as a task-based model that operationalizes corpus-derived linguistic patterns. GSIC transforms diplomatic discourse into classroom practice, enabling grammar to function not as isolated form but as a pedagogic vehicle for nurturing sustainability thinking, linguistic accuracy, and global citizenship in African English classrooms.

## 4. Methodology

This study employed a corpus-based, multi-level discourse analysis to investigate how African leaders linguistically construct sustainability priorities in their United Nations General Assembly (UNGA) 2025 speeches (see Supplementary Material 1). and how these linguistic features map onto sustainabililty competencies [35]. The methodological design integrated quantitative tools from corpus linguistics with qualitative discourse analysis to identify grammatical patterns across five linguistic levels and evaluate their functional relationships with sustainability competencies.

### 4.1. Corpus description

The corpus consisted of official speeches delivered by African heads of state and national representatives during the 80th UNGA session (September–October 2025). All texts were delivered in English from 21 African countries, enabling standardized linguistic analysis. The final dataset comprised 50,644 tokens, 1,956 sentences, and 1,515 paragraphs**,** representing high-stakes diplomatic discourse detailing African nations’ developmental priorities. All speeches were publicly delivered during the 80th UNGA session and retrieved from official United Nations documentation repositories and national government websites, ensuring that the corpus represents authentic, completed diplomatic discourse rather than simulated or prospective data.These UNGA speeches were selected because they serve as authoritative articulations of national and continental positions on the Sustainable Development Goals (SDGs), thus providing authentic evidence of how Africa frames sustainability concerns. Their selection also presents strong pedagogical relevance, as such texts offer realistic language input for advanced English learners developing academic, civic, and professional communicative competencies. Transcripts were downloaded from official UN repositories and government sites, cleaned to remove extraneous information, verified against audiovisual records when available, and segmented into sentences and paragraphs using spaCy before linguistic coding.

Because this study analyzes only publicly available official UNGA speeches and government documents, and involves no human participants or personal data, formal ethical approval was not required.

All speeches analyzed in this study were obtained from publicly accessible United Nations documentation repositories and official government websites. The texts are official diplomatic records intended for public dissemination and are available without access restrictions. Data collection and analysis complied with the terms and conditions of the respective sources, and no copyrighted, confidential, or restricted materials were used. The corpus was compiled solely for academic research purposes in accordance with fair use and non-commercial scholarly guidelines.

#### 4.1.1. Competency coding rules.

Competency assignment followed predefined functional coding rules derived from the UNESCO–Wiek framework. Linguistic features were mapped to competencies based on their dominant communicative function in SDG-tagged contexts rather than surface form alone. Specifically:

- **Normative competence** was coded when grammatical forms expressed obligation, value judgment, or ethical necessity (e.g., *must, should*, evaluative lexis).
- **Systems-thinking competence** was coded when structures encoded causal interdependence or systemic relationships (e.g., *if–then* conditionals, passive constructions foregrounding structural agency).
- **Interpersonal competence** was coded when features constructed collective identity or collaborative agency (e.g., *we, our*, inclusive appeals).
- **Anticipatory competence** was coded when future-oriented constructions projected scenarios or consequences (e.g., *will*, future conditionals).
- **Strategic competence** was coded when discourse followed problem–solution sequencing or articulated concrete policy actions.

In cases where a feature could theoretically signal multiple competencies (e.g., *may* expressing either anticipation or normativity), classification was determined by local discourse function and co-text, following qualitative inspection of concordance lines.

### 4.2. Analytical approach

This study adopts a descriptive corpus-linguistic approach. Quantitative results are reported as frequencies, normalized counts, and SDG-context percentages to map linguistic distributions within a bounded corpus, rather than to support inferential or population-level generalizations. The analysis is exploratory in nature and focuses on identifying internal patterning and functional associations within African UNGA discourse, rather than testing statistical significance.

#### 4.2.1. Operational definition of sustainability discourse.

To ensure conceptual and methodological clarity, sustainability discourse in this study was operationally defined using a lexically anchored procedure derived from official United Nations Sustainable Development Goal (SDG) documentation. A comprehensive SDG lexicon was constructed based on the UN SDG framework, incorporating goal-specific terminology (e.g., *poverty, climate, justice, innovation*), sustainability-related semantic fields (e.g., *sustainable, resilience, equity, transition*), and associated environmental, social, and governance vocabulary explicitly referenced in UN policy documents.

At the preprocessing stage, each sentence in the corpus was automatically tagged using Python-based pattern matching to determine the presence or absence of SDG-aligned lexical items. A sentence was classified as sustainability discourse only if it contained at least one SDG lexicon item. Sentences lacking such markers were excluded from sustainability analysis to prevent conflation with general diplomatic, institutional, or developmental rhetoric.

All subsequent grammatical, pragmatic, and discourse-level analyses were conducted exclusively within these SDG-tagged sentences. This two-stage analytical procedure— [1] lexical anchoring and [2] functional linguistic analysis—ensured that sustainability was methodologically identified prior to interpretation rather than inferred post hoc. By distinguishing SDG-specific discourse from generic political language, the study establishes a transparent operational boundary between sustainability discourse and broader institutional communication.

The SDG lexicon comprised 327 items derived from official UN SDG documentation, including goal-specific keywords (e.g., *poverty, hunger, climate, justice, innovation*), sustainability fields (*sustainable, resilience, transition, equity*), and multiword expressions (e.g., *climate change*, *gender equality*). Polysemous terms such as *development*, *growth*, and *justice* were retained only when occurring in proximity to SDG-aligned semantic fields. A sentence was classified as SDG-related if it contained at least one lexicon item. The full SDG lexical list (see Supplementary Material 2 – S2 in S1 Text) and representative tagging examples are provided in Supplementary Materials.

#### 4.2.2. Multi-level linguistic analysis.

In this study, linguistic analysis was organized across five explicitly defined levels. The *lexical level* examined SDG-aligned content words (nouns, verbs, adjectives) drawn from a curated SDG lexicon, excluding function words, with multiword expressions (e.g., *climate change*, *gender equality*) treated as unified semantic units. The *morphological level* focused on derivational patterns and sustainability-related root morphemes (e.g., *develop-*, *sustain-*, *renew-*), rather than inflectional variation. The *syntactic level* analyzed structures directly implicated in stance and agency construction—specifically modal verbs, conditional clauses, passive constructions, and subordination—selected for their documented relevance to obligation, causality, and responsibility. The *pragmatic level* examined persuasive functions including urgency markers, collective appeals, imperatives, and hedging devices. Finally, the *discourse level* captured macro-organizational patterns such as problem–solution sequencing and cohesion markers (additive, causal, adversative, temporal), identified through manual coding and supported by automated extraction. These levels were analytically distinct but interpreted relationally to capture how sustainability meaning emerges across grammatical and rhetorical strata.

SDG tagging was operationalized through exact, lemmatized, and multiword expression matching against a curated SDG lexicon derived from official UN documentation. Sentences were classified as sustainability discourse only when containing at least one SDG-aligned lexical item; sentences without such markers were excluded from sustainability analysis.

A five-level analytical framework was adopted to capture the structural and functional dimensions of sustainability discourse across lexical, morphological, syntactic, pragmatic, and discourse levels. Following SDG-context identification, lexical analysis was conducted using AntConc to extract SDG-specific terms and determine which goals received the highest frequency representation. Morphological analysis examined derivational patterns, initially focusing on affixation before shifting toward root morphemes (e.g., *develop-*, *renew-*, *sustain-*) once it became evident that sustainability semantics were concentrated in root families rather than affixes alone.

At the syntactic level, modal verbs (*must, will, should, may, might, could*) were extracted and classified according to semantic force and SDG context, while conditional structures, passive voice, and clausal subordination were parsed using spaCy dependency models. Pragmatic analysis examined persuasive strategies through the identification of urgency markers, collective appeals, and imperatives, alongside hedging devices such as weak modals and epistemic markers. Finally, discourse-level analysis coded macro-organizational structures, including problem–evidence–solution schemas and cohesion markers, classified by function (additive, adversative, causal, temporal).

Each linguistic feature was analyzed both quantitatively (frequency counts, normalized distributions, SDG-context percentages) and qualitatively (functional interpretation in context), enabling a multidimensional characterization of how African leaders linguistically construct sustainability meaning.

Lexical SDG frequency calculations were based exclusively on content words (nouns, verbs, adjectives) drawn from the curated SDG lexicon. Function words (e.g., determiners, auxiliaries, prepositions) were excluded from lexical counts. Multiword SDG expressions (e.g., *climate change*, *gender equality*, *human rights*) were treated as unified semantic units where applicable. These decisions ensured that frequency distributions reflected thematic sustainability content rather than grammatical scaffolding.

#### 4.2.3. Coding procedures.

For SDG context identification, inclusion criteria required the presence of at least one SDG-aligned lexical item from the curated SDG lexicon within a sentence. Sentences containing such items were coded as sustainability discourse, while sentences lacking SDG markers were excluded from sustainability analysis to avoid conflation with general diplomatic or institutional language. Automated tagging was performed using Python-based pattern matching across the full corpus. Quantitative feature extraction was conducted using AntConc and Python (spaCy), which supported part-of-speech tagging, dependency parsing, lemmatization, and pattern matching. Custom Python scripts were used to compute SDG context percentages for all linguistic feature classes. Qualitative coding focused on features requiring contextual interpretation, including argument staging, pragmatic intention, and SDG relevance.

To validate tagging accuracy and qualitative coding, two coders independently annotated 20% of the corpus. Inter-rater reliability was calculated using Cohen’s kappa, achieving strong agreement across primary categories (argument stage κ = .81; pragmatic function κ = .85; SDG relevance κ = .92), indicating high coding consistency.

Automated and manual outputs were subsequently integrated into structured datasets in CSV and JSON formats linking linguistic features to SDG categories and discourse functions. These datasets were visualized using Python (matplotlib/seaborn) to represent distributional patterns across linguistic levels.

All texts were lowercased prior to processing. Punctuation was retained for sentence segmentation but excluded from frequency calculations. Tokens were lemmatized to collapse inflectional variants (e.g., *develop*, *developed*, *developing*). Duplicate procedural sentences introduced during UN protocol were removed. Frequency values were normalized per 10,000 tokens to enable cross-feature comparison.

#### 4.2.4. Replication materials and data outputs.

To enhance transparency and replicability, all intermediate datasets and analytical outputs are provided as Supplementary Materials. These include the cleaned UNGA corpus, sentence-level SDG-tagged CSV and JSON files, feature-frequency tables, and concordance exports for key grammatical features (modal verbs, collective pronouns, conditionals, and problem–solution markers) (see Supplementary Material 5 - S5 in S1 Text). A Token Processing Documentation (see Supplementary Material 4 – S4 in S1 Text). file details tokenization, lemmatization, normalization procedures (per 10,000 tokens), handling of protocol repetitions, and filtering decisions. A Methods Summary document provides step-by-step instructions for reproducing SDG tagging, feature extraction, and competency mapping using Python (spaCy 3.7) and AntConc 4.2.0.

### 4.3. Development of mapping to sustainability competencies

The mapping of linguistic features to sustainability competencies was guided by the UNESCO Education for Sustainable Development framework and the sustainability competency model [35]. This identifies systems-thinking, anticipatory, normative, strategic, and interpersonal competencies as core dimensions of sustainability learning. First, functional analysis determined how grammatical features contributed to sustainability meaning: modal verbs (must) signaled ethical obligation (normative competence), conditional structures (if…then) and passive constructions emphasized systemic causality (systems-thinking competence), collective pronouns (we, our) constructed shared agency (interpersonal competence), and future-oriented forms (will) projected scenario planning (anticipatory competence). Second, empirical validation using SDG context percentages confirmed these associations—for example, over 72% of modal verbs occurred in sustainability contexts, and more than 80% of collective pronouns aligned with SDG discourse, supporting their interpersonal function. Third, quantitative aggregation summarized how features distributed across the competency framework, generating a linguistic–competency map indicating which grammatical levels (lexical, morphological, syntactic, pragmatic, discourse) contributed most strongly to each competency. These findings provided the empirical foundation for the development of the Grammar-for-Sustainability Instructional Cycle (GSIC) as a pedagogical application of linguistic–competency alignment.

SDG-context percentages were not interpreted as competency indicators in isolation; rather, they served as empirical validation of function-based mappings. High SDG alignment confirmed that features such as modal obligation, collective pronouns, and conditional reasoning were systematically deployed within sustainability discourse rather than general diplomacy. Competency attribution was therefore grounded in a two-stage process: (1) qualitative functional analysis of linguistic role, followed by (2) quantitative confirmation that the feature operated predominantly in SDG contexts. This ensured that competency mappings reflected communicative purpose rather than raw frequency alone.

### 4.4. Model development procedure

The Grammar-for-Sustainability Instructional Cycle (GSIC) was developed through a sequential process informed by the results of RO1 (grammatical feature identification) and RO2 (competency mapping). First, a corpus-linguistic analysis of UNGA 2025 speeches by African leaders was conducted to identify salient grammatical patterns related to sustainability discourse across five linguistic levels—lexical, morphological, syntactic, pragmatic, and discourse. Findings revealed that sustainability meaning was constructed predominantly through lexical resources (48.6%) and discourse organization (26.5%), supported by persuasive pragmatic strategies (16.5%) such as collective appeals, urgency markers, and intensifiers. Syntactic and morphological features—particularly modal verbs, conditionality, passive constructions, and sustainability morphemes (develop-, sustain-, renew-)—also played important functional roles.

Next, these linguistic features were systematically mapped to sustainability competencies. Results demonstrated clear functional correspondences: modal obligation (must, should) to normative competence; conditional and passive structures to systems-thinking; future projection to anticipatory competence; collective pronouns (we, our) to interpersonal competence; and problem–solution / additive cohesion patterns to strategic competence. This mapping provided the pedagogical rationale for treating grammar as a conduit of sustainability meaning rather than isolated structure.

Drawing on these empirical associations, the GSIC model was designed as a five-phase task cycle: (1) Input & Noticing, where students examine corpus-derived concordance lines; (2) Functional Analysis, comparing grammatical choices and their rhetorical force; (3) Transformation, where learners reformulate authentic UNGA statements to manipulate obligation, agency, and stance; (4) Output & Production, in which learners produce SDG-aligned texts (e.g., mini-UN speeches) modeled on corpus patterns; and (5) Reflection & Evaluation, where learners justify linguistic choices by referencing corpus evidence and sustainability intent. The cycle emphasizes iterative movement between corpus evidence and productive use, enabling learners to understand and apply grammatical choices as purposeful social action. The resulting instructional model operationalizes empirical findings into a task-based scaffold that measurably supports both grammatical development and the cultivation of sustainability competencies.

GSIC is designed for upper-intermediate to advanced secondary and tertiary EFL learners, particularly within African educational contexts where English functions as a medium for academic and civic engagement. Because UNGA texts involve formal register and abstract policy language, learners are expected to possess basic argumentative writing skills and familiarity with modal verbs and complex sentences. While the model was developed using digital corpus tools, GSIC can be implemented in low-resource classrooms through printed concordance lines, teacher-curated excerpts, and pre-annotated worksheets. Teachers require introductory training in corpus-informed pedagogy and functional grammar, and a typical GSIC cycle can be implemented over two to four class sessions depending on depth. For blended or online environments, concordance activities and production tasks can be conducted using shared documents or learning management systems, allowing GSIC to remain adaptable across delivery modes.

## 5. Results

The results are presented by linguistic level (lexical, morphological, syntactic, pragmatic, discourse), following the operational definitions outlined in Section 4.2.2. For each level, quantitative distributions are triangulated with representative corpus excerpts (Section 5.7) to illustrate how grammatical features function within SDG-tagged contexts. All SDG-context percentages are calculated relative to total occurrences of each feature class in the corpus (e.g., percentage of all modal verbs occurring in SDG-tagged sentences). Apparent near-zero values in some figures (e.g., morphology) reflect visualization scaling rather than absence of function; qualitative analysis demonstrates that core morphemes (e.g., *develop-*, *sustain-*) carry high semantic load despite lower surface frequency.

### 5.1. Lexical level

**Fig 1:**
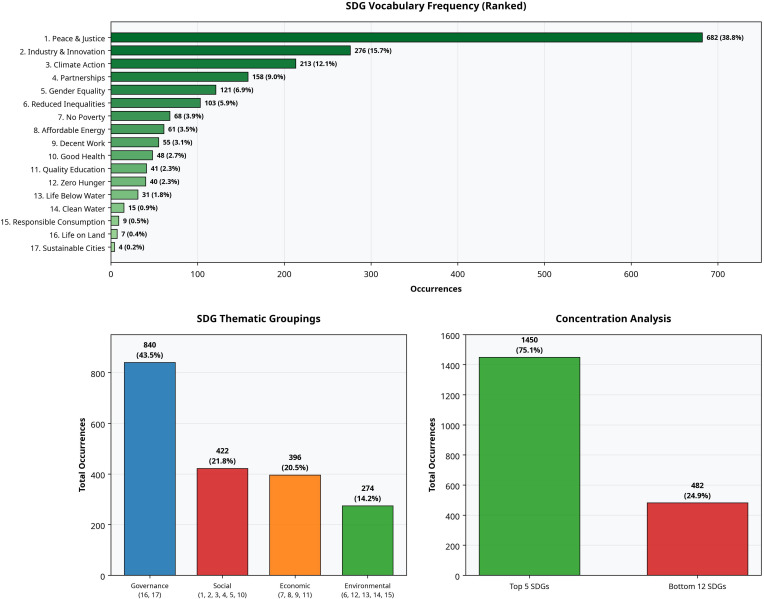
SDG Vocabulary Distribution Across All 17 Goals (Lexical Level).

Analysis of SDG-related vocabulary in African leaders’ UNGA-2025 speeches (Fig 1) reveals a highly concentrated sustainability discourse dominated by governance-oriented themes. SDG 16 (Peace, Justice, and Strong Institutions) accounts for nearly 39% of all SDG lexical items, with SDG 17 (Partnerships) extending this governance emphasis to 43.5% of total frequency, indicating that leaders conceptualize sustainability primarily as an institutional, security, and human-rights challenge. Strong representation of SDG 9 (Industry & Innovation) and SDG 13 (Climate Action) demonstrates a developmental-technological orientation, where sustainable growth and climate response are framed as mutually reinforcing priorities. Social well-being themes—gender equality, inequality reduction, poverty, education, and health—comprise just over one-fifth of total references, suggesting a secondary but meaningful commitment to distributive justice. Conversely, ecological sustainability (SDGs 14, 15, 11, 12) is marginal, representing fewer than 3% of lexical items, illustrating limited attention to biodiversity, ecosystems, and sustainable cities. The top five SDGs constitute over 75% of all lexical frequency, confirming a selective SDG focus anchored in governance, development, and climate concerns rather than comprehensive cross-goal treatment.

### 5.2. Morphological level

**Fig 2:**
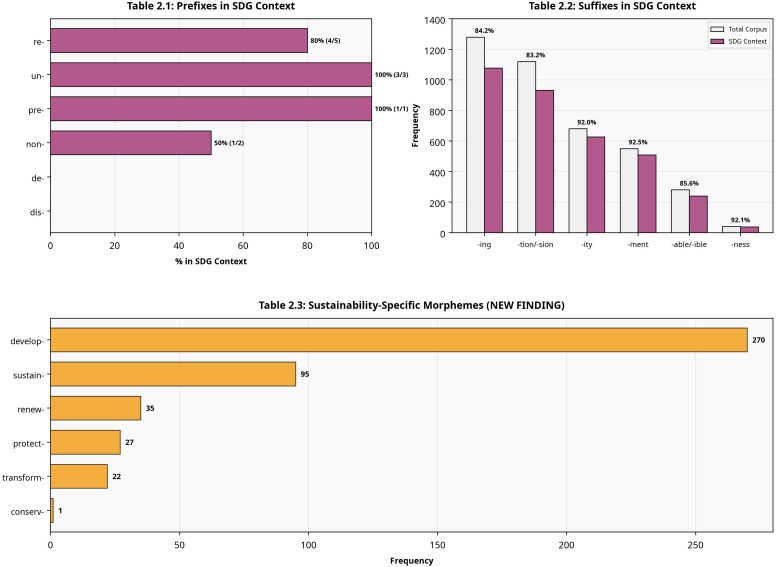
Morphological-Level Analysis of Sustainability Discourse (Percentages indicate proportion of each morphological feature occurring within SDG-tagged sentences relative to total corpus occurrences. Apparent near-zero values reflect visualization scaling; low frequency does not imply low semantic importance).

Morphological analysis revealed that sustainability discourse in African UNGA-2025 speeches is strongly characterized by nominalization and development-centered root morphemes. As shown in Fig 2, more than 83–93% of all major suffixes (-ing, -tion/-sion, -ity, -ment, -able/-ible, -ness) appeared within SDG-related contexts, highlighting a tendency to express sustainability concepts through abstract nouns such as *development, sustainability, equality,* and *empowerment*. This heavy nominalization, particularly in the high frequencies of -tion/-sion and -ment forms, frames sustainability as an institutional and systemic phenomenon rather than a set of discrete individual actions. Likewise, the dominance of -ing forms reflects a focus on ongoing processes (e.g., *developing, building, implementing*), reinforcing a vision of sustainability as continual transformation. Sustainability-specific root morphemes further underscore this orientation: **develop-** (270 tokens) overwhelmingly leads the lexeme field, followed by **sustain**- (95), **renew**- [35], and **protect-/transform**- (27/22). This ordering reveals a distinctly developmental and transformative framing of sustainability, with conservation-based morphemes (e.g., *conserv*-, 1 token) receiving minimal attention. Overall, the morphological profile shows that African leaders construct sustainability as an active, future-focused development agenda grounded in systemic reform and long-term institutional processes.

### 5.3. Syntactic level

**Fig 3:**
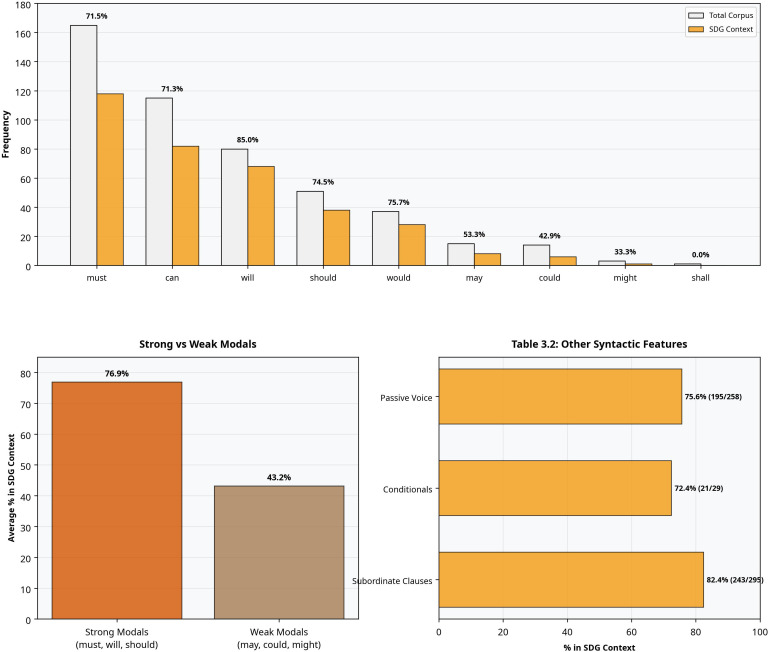
Syntactic-Level Analysis of Sustainability Discourse.

Syntactic analysis shows that African leaders’ UNGA-2025 sustainability discourse is strongly shaped by modal and complex clause structures that foreground obligation, commitment, and systemic reasoning (Fig 3). Over 72% of modal verb occurrences appear in SDG-related contexts, with strong modals—*will, would, should,* and especially *must*—substantially outnumbering weak modals, underscoring a rhetorical stance of urgency and moral necessity rather than speculation. This preference constructs sustainability as non-negotiable and action-oriented. Supporting this assertive stance, passive constructions (75.6%) recast agency from individuals to broader institutions or collective actors, reinforcing sustainability as a systemic rather than personal imperative. Conditional forms (72.4%) frequently link present action to future consequence, embedding causal logic that promotes anticipatory reasoning. Further, subordinate clauses (82.4%) introduce argumentative depth, enabling leaders to articulate complex, interdependent policy narratives. Collectively, these syntactic patterns indicate that sustainability is framed as an urgent, systemic, and forward-looking agenda requiring coordinated, ethically grounded action.

### 5.4. Pragmatic level

**Fig 4:**
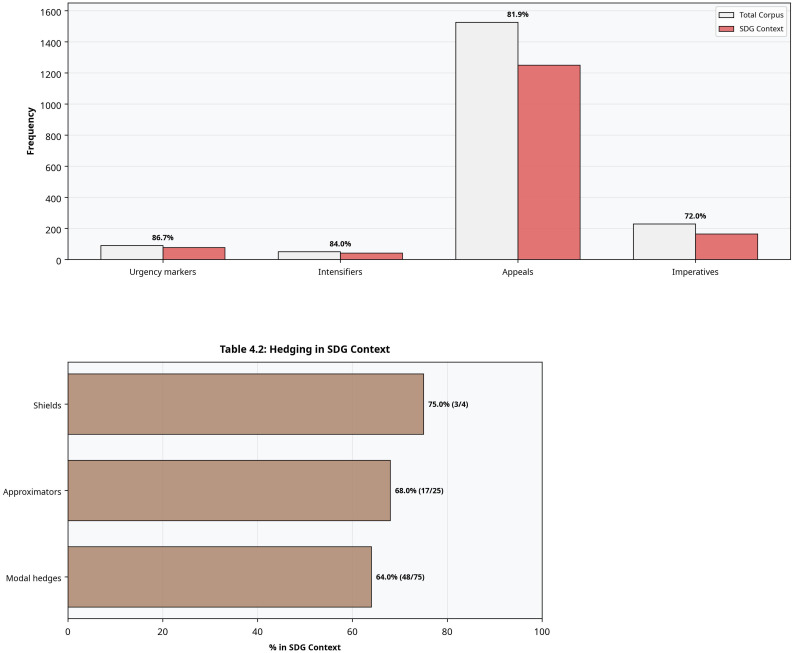
Pragmatic-Level Features in Sustainability Discourse.

Pragmatic analysis shows that sustainability discourse in African UNGA-2025 speeches is overwhelmingly assertive and action-oriented, driven by strong persuasive force rather than diplomatic restraint. Persuasion strategies—including urgency markers (86.7%), intensifiers (84.0%), collective appeals (81.9%), and imperatives (72.0%)—appear predominantly in SDG-related contexts, indicating that sustainability is framed as an immediate, collective, and non-negotiable priority (Fig 4). Urgency and intensification heighten the perceived severity of climate and development challenges, while collective appeals recast sustainability as a shared global responsibility, reinforcing solidarity and multilateralism. Imperative forms further position leaders as norm advocates pressing for decisive global action. Although hedging is present, it is comparatively rare; only 64–75% of hedging occurs within SDG discourse, producing a persuasion-to-hedging ratio of approximately 18:1. This imbalance indicates a rhetorical style that privileges conviction, clarity, and moral pressure, deploying hedging sparingly to acknowledge uncertainty without diluting the urgent sustainability mandate. Overall, pragmatic patterns construct sustainability as a crisis requiring immediate, coordinated, and assertive global response.

### 5.5. Discourse-level

**Fig 5:**
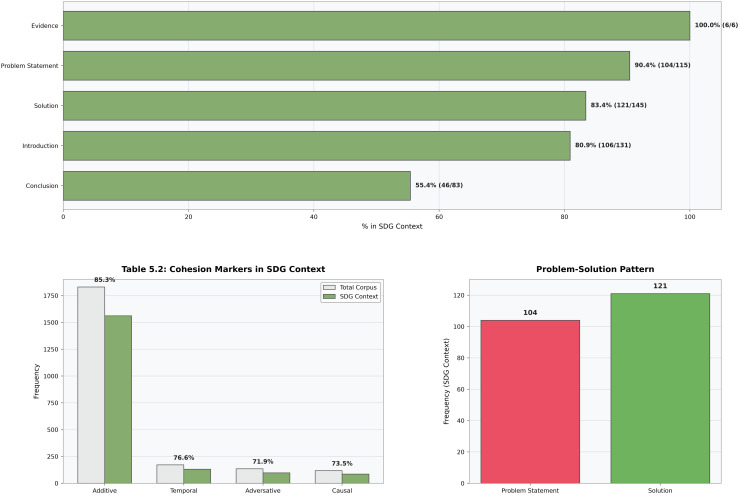
Discourse-Level Features in Sustainability Discourse.

Discourse-level analysis (Fig 5) indicates that African UNGA-2025 speeches consistently structure sustainability through problem–solution argumentation supported by cumulative cohesion. Argument staging shows that problem statements (90.4%), solutions (83.4%), and especially evidence (100%) appear overwhelmingly in SDG contexts, demonstrating that sustainability constitutes the primary domain in which leaders articulate challenges, cite data, and propose interventions. This pattern highlights sustainability as the central policy problem of the era and positions African nations as proactive agents offering actionable responses—often focused on finance, technology transfer, and capacity building. The slight surplus of solutions over problem statements suggests an ultimately hopeful narrative emphasizing agency. Cohesion markers are similarly SDG-concentrated, especially additive forms (85.3%), which cumulatively build multi-dimensional sustainability narratives; temporal, causal, and adversative markers reinforce urgency, causality, and balanced reasoning. Together, these discourse strategies construct sustainability as a complex global challenge requiring coordinated, evidence-based solutions and sustained multilateral engagement.

### 5.6. Linguistic levels distribution

**Fig 6:**
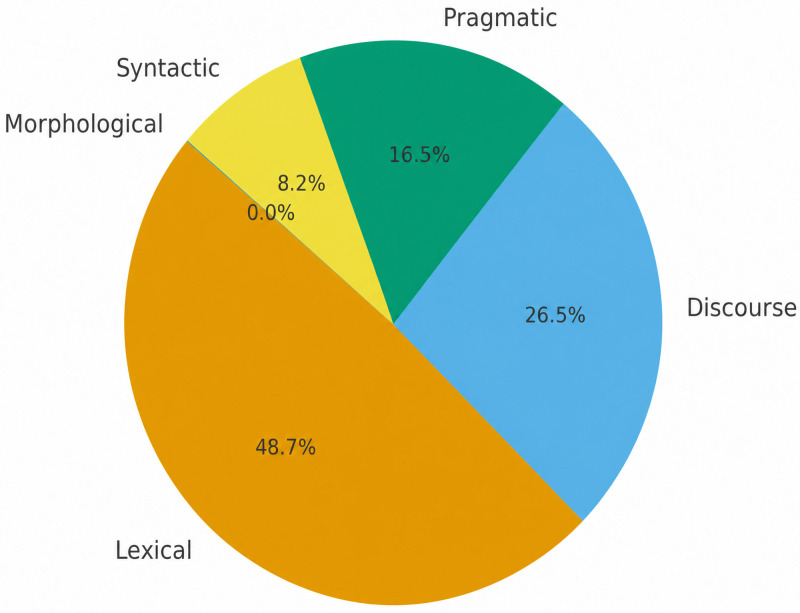
Feature Distribution Across Linguistic Levels (Linguistic level proportions are based on relative frequency of coded features; visual scaling may underrepresent low-frequency but semantically central features such as sustainability morphemes).

The distribution of grammatical features across linguistic levels demonstrates that sustainability discourse in African UNGA-2025 speeches is overwhelmingly lexical in nature, with nearly half of all features (48.6%) expressed through vocabulary choice (Fig 6). This confirms that sustainability meaning is primarily constructed through key thematic terms—such as those linked to governance, development, and climate—rather than through structural or interactional forms alone. Discourse-level features (26.5%) constitute the second-largest category, indicating that sustainability communication also depends heavily on how ideas are organized through problem–solution staging and cohesive argumentation. Pragmatic strategies (16.5%) further reveal the prominence of persuasive appeals and collective mobilization, reinforcing sustainability as an advocacy-driven communicative act. Syntactic structures (8.2%), including modal verbs and conditional clauses, appear less frequently but provide essential nuance by expressing obligation, possibility, and causal reasoning. Although least frequent, morphological features (≈0% visually; but qualitatively notable through core morphemes such as *develop-* and *sustain-*) highlight the semantic consolidation of development-centered discourse. Overall, the results indicate that lexical selection and discourse organization form the foundation of sustainability expression, supported by persuasive pragmatics and syntactic reasoning.

### 5.7. Mapping linguistic features to UNESCO sustainability competencies

**Fig 7:**
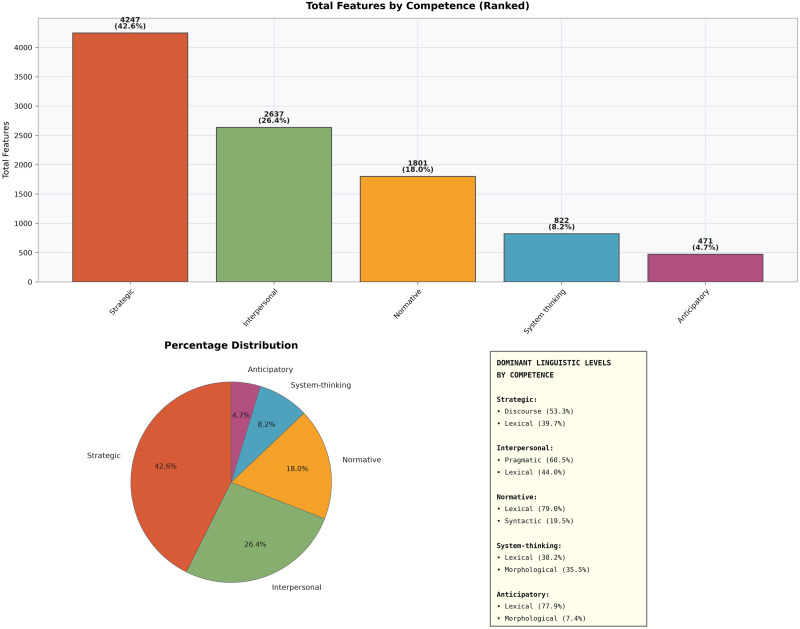
Distribution of Linguistic Features Mapped to Sustainability Competencies.

The competence-level analysis shows that sustainability expression in African UNGA-2025 speeches is dominated by strategic (42.6%) and interpersonal (26.4%) competence features, demonstrating that leaders primarily frame sustainability through coalition-building, negotiation, and multilateral engagement (Fig 7). Strategic competence relies heavily on discourse structures (53.3%), including problem–solution sequencing and cohesion markers, indicating that sustainability claims are communicated through complex, logically organized argumentation rather than isolated statements. Interpersonal competence is characterized by pragmatic strategies (60.5%) such as solidarity appeals and collective pronouns, reflecting an emphasis on shared responsibility and cooperative action. Normative competence (18.0%)—driven almost entirely by lexical values language (79.0%)—centers on peace, justice (SDG 16), equality (SDG 5), and human rights (SDG 10), confirming a strong ethical grounding in sustainability discourse. System-thinking competence (8.2%) shows the most balanced linguistic profile, combining lexical, morphological, and syntactic features to highlight development-oriented systems and causal interdependence. Finally, anticipatory competence (4.7%) is expressed primarily through future-oriented lexical forms and renew-based morphology, underscoring a climate-focused projection of risks and solutions. Collectively, these patterns reveal a discourse in which sustainability is framed foremost as a strategic and collaborative undertaking anchored in ethical commitments, supported by systemic reasoning, and projected through future-oriented climate narratives.

**Fig 8:**
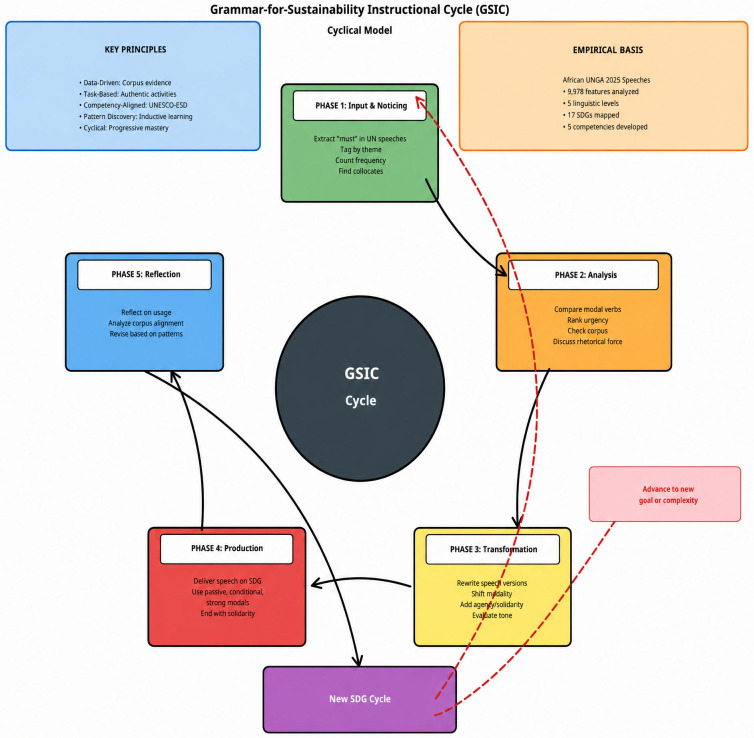
Grammar-for-Sustainability Instructional Cycle (GSIC) Model.

The Grammar-for-Sustainability Instructional Cycle (GSIC**) in** Fig 8 is a data-driven, task-based pedagogical model that integrates authentic UNGA speech corpus with SDG content to develop linguistic and sustainability competencies simultaneously. Its design is grounded in empirical findings demonstrating that African leaders construct sustainability discourse primarily through lexical (48.6%) and discourse-level (26.5%) resources, supported by pragmatic persuasion strategies (16.5%) such as collective appeals, urgency markers, and intensifiers. Results further showed that grammatical forms directly mapped to sustainability competencies: normative competence was expressed through modal verbs signaling obligation (“must,” “should”); systems-thinking competence through conditional and passive structures highlighting interdependence; interpersonal competence through collective pronouns (“we,” “our”); anticipatory competence through future-oriented structures; and strategic competence through problem-solution and additive cohesion patterns. The prominence of SDG-aligned vocabulary—particularly SDG-16 (peace & justice), SDG-9 (industry & innovation), and SDG-13 (climate action)—demonstrates that sustainability discourse is lexicalized around justice, development, and climate futures. These findings collectively justify GSIC’s focus on functional grammar, because grammatical choices are empirically shown to carry ethical positioning, collective agency, and action orientation in real-world sustainability communication.

### 5.8. Representative corpus excerpts across linguistic levels

To enhance interpretability and enable independent verification of quantitative patterns presented in Figs 3–7, representative corpus excerpts were selected from the UNGA 2025 African leaders’ speech corpus (see Supplementary Material 3 – S3 in S1 Text). These examples illustrate how key grammatical features function in sustainability discourse and complement the frequency-based visualizations across syntactic, pragmatic, and discourse levels.

As shown in Fig 3, strong modal verbs (particularly *must* and *will*) occur predominantly in SDG contexts, encoding obligation and ethical necessity. This pattern is exemplified in the following excerpt:

#### 5.8.1. Modal obligation (Normative competence; Fig 3).

*" The UN must now focus on supporting the building of economies of the world to address issues of poverty and the global financial crisis”* (UNGA 2025, Eswatini)

Here, *must* constructs sustainability as a moral imperative rather than optional policy, operationalizing normative competence through linguistic obligation.

Fig 4 demonstrates high concentrations of collective appeals (we, our) in sustainability discourse. This interpersonal framing is reflected in:

#### 5.8.2. Collective pronouns (Interpersonal competence; Fig 4).

*“It is essential that we address the climate crisis as the greatest existential threat of our time, against which we can only achieve results if we act together, in a coordinated and solidarity - based manner”* (UNGA 2025, Angola)

The repeated inclusive pronouns linguistically construct shared agency and solidarity, enacting interpersonal competence by positioning sustainability as a collective responsibility.

Conditional structures highlighted in Fig 3 model causal relationships between present action and future consequences:

#### 5.8.3. Conditional reasoning (Systems-thinking competence; Fig 3).

*“If this were indeed true, a continent as large and with as many UN Member states as Africa would have at least one permanent seat on the Security Council”* (UNGA 2025, Ghana)

This if–then configuration explicitly encodes a causal relationship between Africa’s size and representation and its exclusion from permanent Security Council membership, thereby realizing systems-thinking competence through structured reasoning about global governance inequities.

#### 5.8.4. Problem–solution discourse (Strategic competence; Fig 6).

As illustrated in Fig 6, sustainability arguments frequently follow a problem–solution sequence:

*PROBLEM: “Climate change is not a distant threat; it is a present reality.”*

*SOLUTION: “That is why I have joined the Global Center on Adaptation, because adaptation must be at the centre of sustainable development.”* (UNGA 2025, Botswana)

This structured progression from identifying climate change as an immediate crisis to committing to institutional adaptation action exemplifies strategic competence by linguistically organizing policy commitment and coordinated response within a sustainable development framework.

Together, these excerpts triangulate the quantitative distributions presented in Figs 3–7 with qualitative evidence from authentic UNGA discourse. They demonstrate that sustainability competencies are explicitly realized through grammar and discourse rather than inferred abstractly, confirming that African leaders systematically mobilize linguistic resources to construct urgency, agency, and coordinated SDG action.

#### 5.8.5. Teacher manual.

As shown in Fig 8, the Grammar-for-Sustainability Instructional Cycle (GSIC) guides teachers in using authentic UNGA-2025 African leaders’ speeches to teach grammar as a tool for sustainability reasoning. Instruction begins with:

### 5.9. PHASE 1 — Input & Noticing

In this introductory stage, the teacher selects excerpts from African UNGA-2025 speeches and provides students with concordance lines that highlight a target grammatical feature (e.g., must, will, if, we). Students read short authentic lines, sort them by SDG themes (e.g., SDG 13 Climate Action, SDG 16 Peace & Justice), and notice patterns—such as how must frequently accompanies climate, peace, and development contexts. The teacher guides students to count frequency, identify collocates (e.g., must + act / must + ensure), and discuss initial interpretations. The teacher may prompt noticing with questions such as: What SDG themes appear most often with must? or Who is presented as responsible (we, global community, government)? This phase concludes with students highlighting emerging meaning–form relationships—for example, must expresses non-negotiable obligation, we constructs collective responsibility, etc. Materials may include printed concordance lines, color-coding tasks, or digital corpus tools, depending on classroom resources.

### 5.10. PHASE 2 — Analysis

Students now examine how different grammatical structures influence meaning and rhetorical strength. The teacher provides parallel examples (e.g., we must transition… / we should transition… / we could transition…) and asks students to rank which sounds most urgent or persuasive. Students analyze modal strength, agency distribution, tone, and audience impact. In small groups, they compare examples from across speeches, noting when leaders choose passive constructions (measures must be taken) or active (we will act), and why. Learners also evaluate pragmatic features such as urgency markers (now, immediately), solidarity forms (our children, our continent), and causal connectives (therefore, because, so that). The teacher facilitates discussion linking grammar to communicative purpose, helping students conclude that grammatical choices are not neutral—they strategically express stance, obligation, and shared responsibility.

### 5.11. PHASE 3 — Transformation

In this phase, students manipulate authentic SDG sentences to explore how small grammatical adjustments impact meaning. The teacher provides real UNGA sentences and asks students to rewrite them by (a) shifting modality (should → must; will → might), (b) changing agency (active ↔ passive), (c) adding collective pronouns (we / our), or (d) modifying tone (from neutral → persuasive). Students compare their revised versions with originals to evaluate how meaning and responsibility shift. For example:

- Original: We should invest in renewable energy.
- Transformed: We must urgently invest in renewable energy to protect our children’s future.

The teacher highlights how transformation processes relate to sustainability competencies—e.g., stronger obligation → normative competence; future orientation → anticipatory competence. This phase promotes flexible grammatical control and conceptual precision.

### 5.12. PHASE 4 — Production

Students now produce original SDG-aligned texts modeled on corpus patterns. Guided by the teacher, students plan and deliver mini-UN speeches, policy statements, campaign messages, position paragraphs, or social-impact pitches about a selected SDG (individually or in groups). They are encouraged to integrate modal verbs (must, should), conditional reasoning (if…then), passive framing for systemic causes, collective pronouns for solidarity, and cohesive problem–solution structures. The teacher may provide planning scaffolds—purpose, target audience, SDG theme, problem–solution structure. Peer review follows, with a checklist referencing the linguistic features and SDG competencies targeted. By engaging in authentic communicative tasks, students internalize grammar as persuasive social action rather than mechanical structure.

### 5.13. PHASE 5 — Reflection

Learners evaluate and refine their work by comparing their language choices to corpus findings and the rhetorical patterns of UNGA speeches. The teacher prompts students to examine whether they used strong or weak modality, distributed agency effectively, used collective pronouns to build solidarity, or organized discourse around problem–solution logic. Students explain their choices (written or oral metalinguistic reflection)—e.g., I used must because it expresses strong climate urgency. They may revise speeches to strengthen clarity or SDG alignment. The teacher encourages linking linguistic decision-making to UNESCO sustainability competencies (normative, systems-thinking, strategic, anticipatory, interpersonal), helping students recognize how grammar fosters ethical judgement and sustainability reasoning. This reflective stage reinforces iterative improvement; students then cycle into a new SDG theme with increased sophistication.

*In sum*, GSIC helps teachers integrate meaningful real-world content into grammar teaching, transforming UN speeches into actionable linguistic models. The cycle promotes critical noticing, functional understanding, transformative rewriting, purposeful production, and reflective metalinguistic reasoning—cultivating both grammar mastery and sustainability competencies.

## 6. Discussion

This section interprets the findings in relation to the study’s research questions, relevant literature, and broader educational implications. The results demonstrate how African leaders construct sustainability discourse through layered grammatical and rhetorical choices, how these linguistic patterns correspond to UNESCO ESD competencies, and how the Grammar-for-Sustainability Instructional Cycle (GSIC) translates these insights into pedagogy.

Findings show that African leaders’ UNGA-2025 speeches present sustainability as a strategic and transformative agenda anchored in development, peace, partnerships, and climate futures. The over-representation of SDG 16 (peace and justice), SDG 17 (partnerships), SDG 9 (industry and innovation), and SDG 13 (climate action) suggests that sustainability is framed not only as environmental protection but also as political stabilization, socio-technical advancement, and climate adaptation. This aligns with work demonstrating Africa’s sustainability priorities as intertwined with governance, stability, and technological development [10]. Notably, ecological conservation (SDG 14, SDG 15) received less emphasis, suggesting that geopolitical and infrastructural constraints place development and climate adaptation ahead of biodiversity protection. Such framing is consistent with Agenda 2063’s narrative of prosperity, innovation, and regional integration. Collectively, these discourse patterns signal Africa’s intention to mobilize sustainability for economic transformation, institutional coherence, and planetary justice.

The analysis demonstrates that African sustainability discourse is linguistically constructed through grammar as a meaning-making resource rather than neutral form. Modal verbs (must, should) overwhelmingly appeared in SDG-tagged contexts, encoding obligation and ethical necessity, confirming modality’s role in stance construction [18]. Conditional constructions (if–then) framed interdependence between climate, peace, and development, expressing systemic causality central to sustainability logic. Collective pronouns (we, our) reinforced interpersonal solidarity and shared responsibility, consistent with interpersonal positioning literature [21]. Problem–solution argumentation patterns provided strategic coherence to sustainability messaging. These grammatical strategies reflect broader findings that political discourse employs grammar to persuade, mobilize, and legitimize rather than merely describe [8]. Thus, grammar in African UNGA discourse is purposeful, political, and motivational, constructing sustainability as urgent, shared, and actionable.

The study’s multi-level analysis revealed strong correspondences between grammatical features and sustanability competencies. Modal verbs expressing obligation aligned with normative competence, while conditional and passive structures aligned with systems-thinking competence by foregrounding interdependence among sectors. Collective pronouns enacted interpersonal competence, while future-oriented forms operationalized anticipatory competence through scenario projection. Finally, problem–solution and additive discourse patterns aligned with strategic competence, mirroring planning and intervention logic [15,30,31]. These findings reinforce previous arguments that sustainability competencies are communicatively enacted [13] but extend the literature by empirically specifying the grammatical realizations of these competencies at scale. The results suggest that African UNGA discourse implicitly models the competencies necessary to enact the SDGs, offering a linguistic blueprint for ESD curriculum design.

The functional alignment between grammar and ESD competencies provides a strong foundation for integrating sustainability discourse into English language instruction. African UNGA speeches offer authentic, contextually meaningful texts that can support communicative competence, critical thinking, and sustainability reasoning simultaneously. These findings reinforce scholarship advocating for SDG integration through language curriculum and communicative task design [1,2]. The corpus-based approach complements data-driven learning [25], encouraging learners to discover patterns in authentic discourse while developing pragmatic and metalinguistic awareness. Furthermore, such integration aligns with project-based and interdisciplinary teaching shown effective in Mandarin and English contexts [4]. Overall, the pedagogical implications support re-imagining grammar as a resource for citizenship, global literacy, and sustainability—not rote form.

The Grammar-for-Sustainability Instructional Cycle (GSIC) operationalizes the study’s corpus-derived insights into a five-phase task cycle—input noticing, analysis, transformation, production, and reflection. GSIC translates authentic UNGA patterns into classroom practice, enabling learners to examine concordance lines, analyze rhetorical force, reformulate statements, produce SDG-aligned discourse, and justify linguistic choices. In doing so, it aligns with data-driven learning [36], corpus-assisted pedagogy, and task-based instruction. Importantly, each phase supports the development of sustainbilities competencies: noticing modal obligation fosters normative competence; analyzing conditionality develops systems-thinking; deploying collective pronouns supports interpersonal competence; and composing problem–solution arguments promotes strategic and anticipatory competence. GSIC thus offers a novel pedagogical bridge, transforming abstract sustainability goals into communicative practice and linking grammar instruction directly to social action.

This study advances research in three major ways. First, it addresses the gap in literature linking ESD and grammar pedagogy by providing empirical evidence that linguistic forms can encode sustainability competencies—an area minimally foregrounded in prior ESD studies [15]. Second, it introduces a novel linguistic–competency mapping framework that connects grammatical patterns to sustainability competencies, thereby extending scholarship on the linguistic foundations of sustainability reasoning [13]. Third, the GSIC model offers a scalable pedagogical innovation that localizes ESD within African discourse contexts, supporting decolonial curriculum transformation by centering African voices and sustainability framings. Collectively, these contributions advance theoretical, analytical, and pedagogical discussions at the intersection of corpus linguistics, sustainability, and language education.

The integration of African UNGA discourse into ELT aligns with broader educational priorities such as Agenda 2063, language-in-education policies, and civic engagement. By foregrounding African sustainability narratives, this work supports Pan-African identity development, multilingual inclusion, and decolonial pedagogy [7]. Using diplomatic discourse as curricular text situates English learning within authentic sociopolitical contexts, equipping learners with communicative competencies relevant to climate diplomacy, peacebuilding, and regional integration. Thus, the GSIC framework complements continental goals for human capital development and strengthens education’s role in advancing Africa’s SDG participation and sustainability leadership.

## 7. Limitations and future directions

While this study provides an empirically grounded mapping of grammatical features to sustainability competencies and proposes the Grammar-for-Sustainability Instructional Cycle (GSIC), several limitations must be acknowledged. First, the corpus is restricted to English-language speeches delivered by African leaders during a single UNGA session. This limits linguistic generalizability, as indigenous African languages and informal sustainability registers are not represented, and contextual generalizability, as UNGA discourse reflects elite diplomatic communication rather than grassroots or community-level sustainability practices. Second, although GSIC is theoretically derived from corpus evidence and aligned with established ESD frameworks, it has not yet been empirically implemented or evaluated in classroom settings; consequently, claims regarding its pedagogical impact remain provisional. Third, the UNGA genre is highly formal and politically mediated, which—while valuable for modeling official sustainability positioning—may not fully capture everyday sustainability communication or be equally accessible to all learner populations.

Future research should therefore pilot GSIC in African EFL classrooms to assess its effects on grammatical development and sustainability competencies through mixed-methods classroom studies. Additional work should extend the corpus to include national policy documents, media discourse, youth statements, and multilingual data to capture broader sustainability voices. Cross-regional comparisons and longitudinal designs would further refine the linguistic–competency mapping framework and strengthen the evidence base for grammar-based ESD pedagogy. Finally, Future work will pilot GSIC with upper-intermediate and tertiary EFL learners and evaluate implementation feasibility across low-resource, blended, and online African classroom contexts.

## 8. Conclusion

This study examined how African leaders construct sustainability discourse in UNGA-2025 speeches and demonstrated how these linguistic patterns can inform SDG-aligned language pedagogy. A 50,644-token corpus was analyzed across five linguistic levels—lexical, morphological, syntactic, pragmatic, and discourse—revealing that sustainability meaning is built predominantly through lexical resources (48.6%) and discourse-level organization (26.5%), supported by pragmatic strategies of solidarity and urgency (16.5%). Key grammatical patterns—including modal obligation, conditional reasoning, passive constructions, future-oriented forms, collective pronouns, and problem–solution structures—mapped systematically onto UNESCO sustainability competencies: normative, systems-thinking, interpersonal, anticipatory, and strategic. These insights informed the development of the Grammar-for-Sustainability Instructional Cycle (GSIC), a five-phase model (noticing, analysis, transformation, production, reflection) that operationalizes authentic sustainability discourse into classroom tasks for integrated development of grammatical control and sustainability competencies.

These findings demonstrate that African sustainability discourse is purposeful rather than stylistically neutral, using grammar to frame urgency, interdependence, and collective agency—particularly around SDG 16 (peace & justice), SDG 17 (partnerships), SDG 9 (industry & innovation), and SDG 13 (climate action). The GSIC model offers a practical bridge between corpus evidence and language teaching, positioning grammar as a vehicle for ethical stance and strategic problem-solving.

## Supporting information

S1 Text S1 File. African Leaders UNGA 2025 speeches. S2 File. SDG lexical list. S3 File. Multi-Level Discourse Analysis (Corpus Evidence). S4 File. Token Processing Documentation. S5 File. Multi-Level Grammatical Analysis (Replication material).
(RAR)
